# Mild SARS-CoV-2 maternal infection in mice induces transient offspring neurodevelopmental aberrance

**DOI:** 10.1073/pnas.2518294123

**Published:** 2026-03-18

**Authors:** Wesley Tung, Matthew Yuen, Helen Cai, Hyesun Cho, Peiwen Lu, Harvey J. Kliman, Robert J. Homer, Alexa Herrerias, Nikkita Salla, Arianna Rodriguez Rivera, Yuting Liu, Kartik Pattabiraman, Akiko Iwasaki

**Affiliations:** ^a^Department of Immunobiology, Yale University School of Medicine, New Haven, CT 06520; ^b^Department of Neuroscience, Yale University School of Medicine, New Haven, CT 06520; ^c^Child Study Center, Yale University School of Medicine, New Haven, CT 06520; ^d^Department of Obstetrics, Gynecology, and Reproductive Sciences, Yale University School of Medicine, New Haven, CT 06520; ^e^Department of Pathology, Yale University School of Medicine, New Haven, CT 06520; ^f^Wu Tsai Institute, Yale University, New Haven, CT 06520

**Keywords:** maternal infection, pregnancy, fetal development, central nervous system, animal behavior

## Abstract

Maternal viral infection during pregnancy has been identified as a risk factor for psychiatric disorders and neurodevelopmental abnormalities in offspring. With cumulative SARS-CoV-2 infections now numbering in the hundreds of millions globally, there is a need to evaluate the effects of maternal SARS-CoV-2 infection on offspring brain development and behavior. We developed a mouse model of mild COVID-19 during pregnancy in which SARS-CoV-2 infection is restricted to the respiratory tract. Infected mothers did not show weight loss or changes in litter size, but did exhibit detectable local and systemic immune responses, including placental inflammation. Characterization of the offspring’s cerebral cortex revealed transcriptomic changes in the fetus at E15 and on postnatal day 5 (P5), but no gross alterations in cytoarchitecture, synaptic density, or microglial abundance. We did not detect any significant changes in open-field or novel object recognition tests in P50 offspring born to SARS-CoV-2-infected dams. These findings suggest that mild maternal respiratory SARS-CoV-2 infection induces soluble factors that mediate placental inflammation and transient cerebral cortex alterations in offspring that resolve by later childhood.

## Significance

The rising global numbers of SARS-CoV-2 infections highlight the need to assess potential neurodevelopmental and psychiatric impact in children born to infected mothers. Human cohorts have provided conflicting conclusions, while mouse studies have focused on moderate-to-severe infection despite most infections in pregnant women being mild or asymptomatic. Our study shows that mild, respiratory tract–restricted SARS-CoV-2 infection in pregnant mice was sufficient to cause placental inflammation and transient changes in offspring brain gene expression, without altering gross brain structure or behavior under our experimental conditions. These findings suggest that soluble factors induced by maternal respiratory infection mediate placental inflammation and changes in offspring brain gene expression during the fetal and neonatal periods, which resolve in later childhood.

There are more than 700 million reported cases of SARS-CoV-2 viral infection globally which resulted in the COVID-19 pandemic. Although the chronic sequelae of COVID-19 disease continue to be widely studied in clinical populations, the potential consequences of SARS-CoV-2 infection during pregnancy will not be fully realized for several decades. One key question is the impact of maternal SARS-CoV-2 infection on neonatal health and development. The work of several groups has suggested that maternal SARS-CoV-2 infection during pregnancy can lead to adverse fetal outcomes, such as stillbirth, preterm birth, small size for gestational age, and reduced birth weight (1–7). Furthermore, increased risk for future neuropsychiatric disease remains a potential concern, as mouse models simulating SARS-CoV-2 infection (8–10) and maternal immune activation (11–15) have suggested that maternal infection can lead to behavioral and neuropsychiatric abnormalities in offspring mice. Mechanistically, it has been suggested that circulating cytokines following SARS-CoV-2 infection may cause placental inflammation, a phenomenon observed in several human cohorts (16–20).

Although mouse models suggest a link between maternal SARS-CoV-2 infection and neuropsychiatric abnormalities in offspring, clinical studies investigating potential early-life changes in developmental trajectories have yielded mixed results (21–25). The discrepancy between human and mouse studies may be due to differences in infection severity between human patients and animal subjects. To date, mouse models that have examined the consequences of maternal SARS-CoV-2 infection have recapitulated moderate-to-severe SARS-CoV-2 infection, with significant weight loss and sickness behaviors observed in dams and loss of fetal viability observed in litters (8–10). In cases of human illness, however, severe and moderate disease represent a small fraction, with most infection cases in pregnant women being mild or asymptomatic (26). Thus, the generation of animal models that simulate mild maternal SARS-CoV-2 infection and enable characterization of its effects on offspring will likely yield insights into potential neurodevelopmental and psychiatric sequelae.

In this study, we developed a mouse model of mild SARS-CoV-2 infection during pregnancy to investigate the impact of mild disease on the offspring’s brain development. We examined immune responses in the infected dams and characterized the impact of infection on the placenta. Furthermore, we probed the impact of maternal infection on offspring brain development and behavior.

## Results

### Development of a Mild SARS-CoV-2 Infection Pregnancy Mouse Model.

Previous models to study the impact of maternal infection on neurodevelopment and psychiatric disease have relied on the use of bacterial or viral mimetics, including lipopolysaccharides or poly I:C, that activate innate immune responses, or in vivo infection models that can induce viral illness and severe symptoms, including mortality (8–10, 27, 28). While these models are capable of recapitulating moderate/severe systemic inflammation, we sought to test the impact of mild/asymptomatic infection on fetal and neonatal neurodevelopment, as SARS-CoV-2 infections in pregnant women primarily entailed mild/asymptomatic cases.

We therefore developed a pregnancy mouse model that mimics mild/asymptomatic viral respiratory infection. Accordingly, we leveraged an existing mouse model developed by our laboratory to study the impact of mild or asymptomatic SARS-CoV-2 infection. In this model, an adeno-associated virus (AAV) expressing human ACE2 (hACE2) is directly delivered to the trachea of wild-type C57BL6/J mice, therefore restricting viral infection and replication to the respiratory tract and lungs. In adult mice, this model was previously shown to recapitulate SARS-CoV-2 immune-related pathology while limiting weight loss and sickness behaviors that can occur because of severe inflammation or direct brain infection (29, 30). Another advantage of this model is that offspring are not directly susceptible to SARS-CoV-2 infection, as neither the dams nor the offspring natively express human ACE2 (31). This is an important feature of our model, as clinical studies have reported minimal vertical transmission of SARS-CoV-2 (32). Furthermore, any observable phenotype in the offspring would be strictly attributable to the dam’s infection and immune response.

**Fig. 1.**
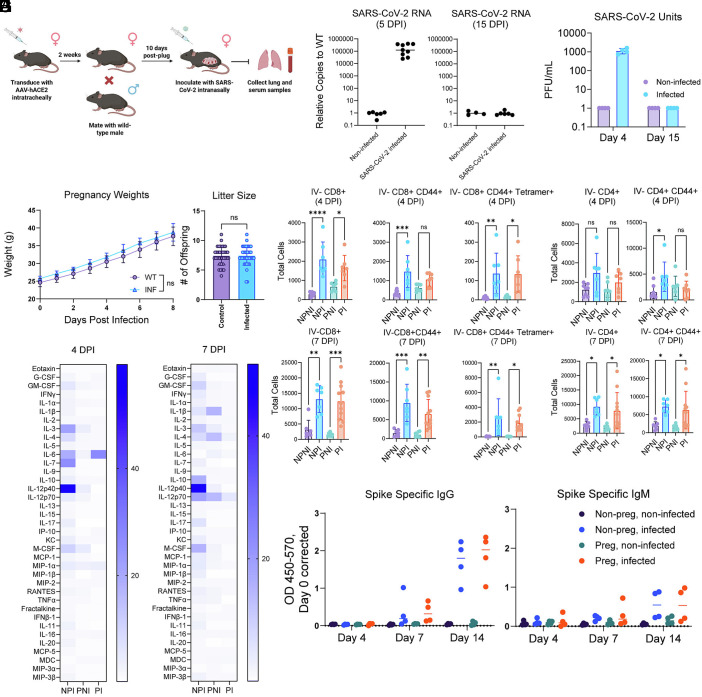
Development of a mild SARS-CoV-2 infection pregnancy mouse model. (*A*) Experimental schematic of mild COVID-19 infection pregnancy model. (*B*) RT-qPCR of SARS-CoV-2 mRNA from whole lung tissue 5 d postinfection (*Left*) or 15 d postinfection (*Right*). (*C*) Plaque assays from lung tissue homogenate. 4 or 15 d postinfection *Left* lobe of the lung was homogenized in PBS prior to quantification of SARS-CoV-2 viral titer. (*D*) Weight of pregnant mice. Control or infected pregnant mice were weighed every day after inoculation at E10. (*E* and *F*) Flow cytometry of T cell subsets in the lung. 4 or 7 d postinfection, the *Right* whole lung was isolated, digested, and subjected to flow cytometry using the described antibodies. NPNI = nonpregnant, noninfected, NPI = nonpregnant infected, PNI = pregnant noninfected, PI = pregnant infected. (*G*) Circulating proinflammatory cytokines. 4- or 7-d postinfection, whole blood was collected retro-orbitally and serum isolated through centrifugation. After incubation with SARS-CoV-2 deactivation solution, serum samples were sent to EveTechnologies for multiplex analysis. (*H*) ELISAs for measurement of anti-SARS-CoV-2 spike protein in serum. 4, 7, and 14 d postinfection, 1:50 dilution of serum was used in conjunction with recombinant SARS-CoV-2 spike protein to detect antibodies against SARS-CoV-2 spike protein. (*B* and *D*–*G*) are compilations of at least 2 independent experiments with at least three mice per experiment. (*C* and *H*) are the results of one experiment with four mice. Statistical analysis was performed using an ordinary one-way ANOVA with multiple comparisons. *P < 0.05, **P < 0.01, ***P < 0.001, and ****P < 0.0001.

In our adapted model, we delivered AAV expressing human ACE2 into the respiratory tract of 8- to 10-week-old C57BL6/J female mice. Two weeks posttransduction, females were paired with wild-type C57BL6/J males, and productive copulation was confirmed, designated as embryonic day (E) 0.5. At E10.5, pregnant dams were intranasally inoculated with a bolus of ancestral SARS-CoV-2 (WA-1) (Fig. 1*A*). In this model, we detected both SARS-CoV-2 RNA and viral replicative units in the lungs of dams at four days post–SARS-CoV-2 infection (Fig. 1 *B* and *C*). Replicative viral units were undetectable by day 15, consistent with our previous studies in nonpregnant mice (29). To assess whether pregnant mice display the same lung pathology as nonpregnant mice in previous reports, we conducted both immunofluorescence staining for SARS-CoV-2 spike protein and hematoxylin and eosin staining for general lung pathology. We found that the SARS-CoV-2 spike protein was readily detectable in dams infected with SARS-CoV-2 compared to mock-infected mice (*SI Appendix*, Fig. S1*A*). In addition, dams infected with SARS-CoV-2 exhibited increased lymphocyte infiltration, similar to that observed in nonpregnant mice in our prior studies (*SI Appendix*, Fig. S1*B*) (29).

To assess the impact of mild COVID-19 infection on pregnancy, we examined weight gain after infection at E10. We observed no difference in postinfection body weight or litter size compared with noninfected pregnant control mice (Fig. 1*D*). Consistent with prior reports, infected pregnant mice did not display external sickness behaviors, including ruffled fur or reduced mobility (29).

To examine the impact of pregnancy on antiviral immune responses, we conducted immunological comparisons of infected pregnant mice and nonpregnant counterparts. In both pregnant and nonpregnant mice, infection led to a comparable increase in total lung-resident CD8+ activated (CD8+ CD44+) and SARS-CoV-2 spike tetramer positive (CD8+ CD44+ Tetramer+) T cells at both 4 d postinfection (DPI) and 7 DPI (Fig. 1*E*). We observed a lack of an activated CD4+ T cell population in pregnant infected mice at 4 DPI, which became comparable to their nonpregnant infected counterparts by 7 DPI (Fig. 1*F*). Together, these results demonstrate that a delayed but detectable immune response was mounted against SARS-CoV-2 in pregnant mice.

To determine the extent and magnitude of the systemic immunological response to SARS-CoV-2 in pregnant mice, we compared the systemic cytokine response between pregnant mice and nonpregnant counterparts at 4 DPI and 7 DPI. At 4 DPI, we noticed a general increase in serum cytokines in both infected pregnant and nonpregnant mice (Fig. 1*H*, *Left*). However, at 7 DPI, we noticed lower serum cytokines in infected pregnant mice compared to infected nonpregnant controls (Fig. 1*H*, *Right*). These results are consistent with prior reports suggesting that pregnancy leads to an immunosuppressive state (33). Finally, we noticed no differences in the capabilities of infected pregnant mice to produce anti-spike IgM and IgG antibodies compared to infected nonpregnant mice (Fig. 1*G*). These data therefore indicate that the antibody response of pregnant mice is comparable to that of nonpregnant mice following SARS-CoV-2 infection, though systemic cytokine responses were reduced in pregnant mice.

### Placentas of SARS-CoV-2-Infected Dams Exhibit Mild Inflammation and Transcriptomic Changes in the Absence of Direct Infection.

Several studies suggest that maternal immune activation from SARS-CoV-2 infection can lead to placental inflammation and influence offspring development (16, 20). Due to the presence of a serum cytokine response in our mild infection mouse model, we hypothesized that those systemic cytokines may induce an inflammatory response in the mouse placenta.

**Fig. 2.**
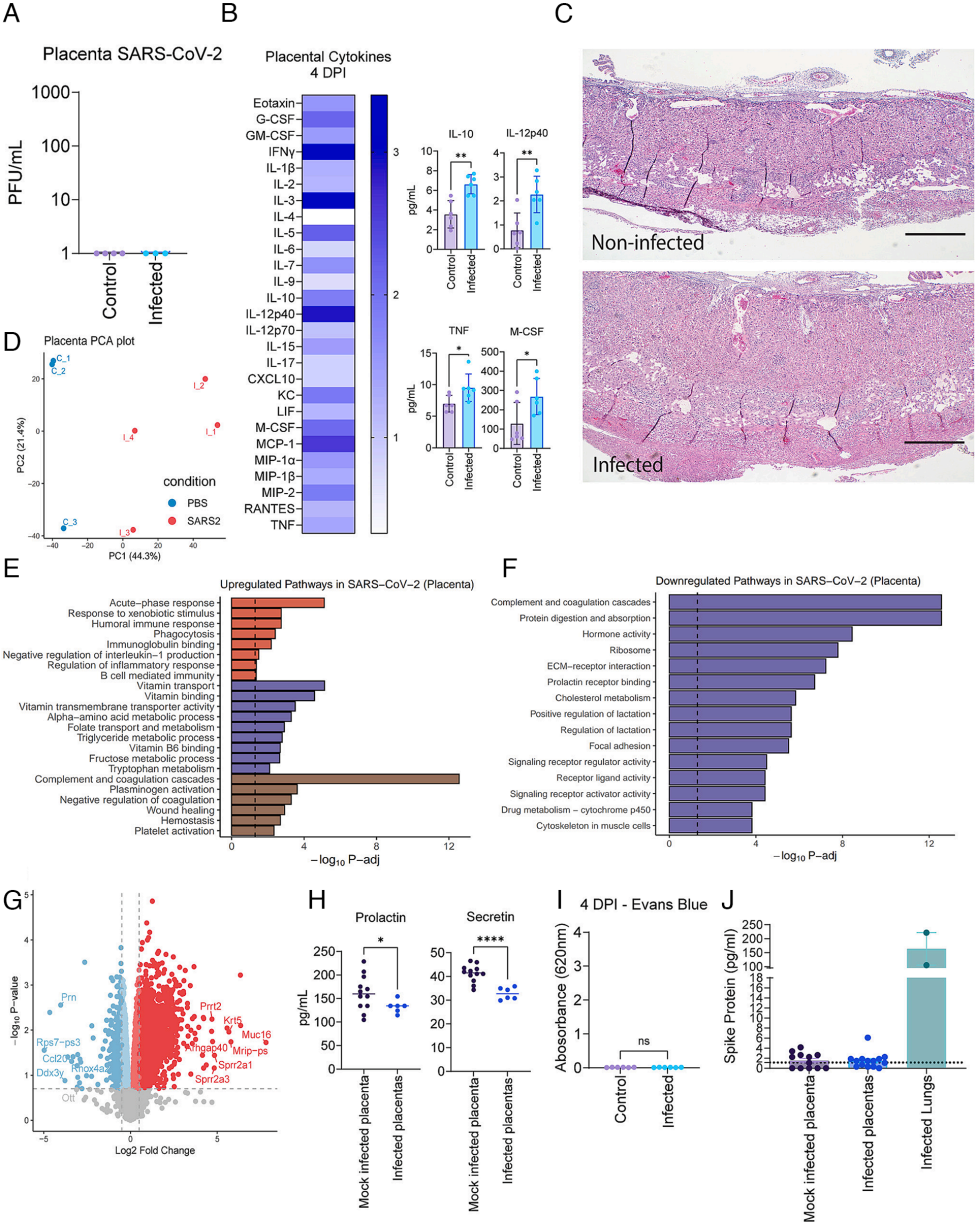
Placentas of SARS-CoV-2-infected dams exhibit mild inflammation and metabolic/hormonal disturbances in the absence of direct infection. (*A*) Plaque assays from placental homogenate collected from dams inoculated with SARS-CoV-2 vs PBS at 4 DPI. (*B*) Heat map of cytokines detected in placentas of dams inoculated with SARS-CoV-2 or PBS at 4 DPI: controls n = 5, infected n = 6. (*C*) Placenta histology. Hematoxylin and eosin staining was conducted on slides derived from placentas collected from dams that were inoculated with SARS-CoV-2 vs PBS at 4 DPI. The Scale bar measures 0.5 mm. 7 dams from both infected and control cohorts were examined. (*D*) Principal coordinate analysis of mRNA isolated from combined whole placentas of infected vs control mothers at 4 DPI; control n = 3, infected n = 4. Each data point represents 3 to 4 placentas pooled from a single unique conceptus. (*E* and *F*) Pathway analysis created by significantly upregulated (*E*) or downregulated (*F*) genes in infected dam placentas, segregated by theme. (*G*) A volcano plot was created using a false discovery rate of 20%. (*H*) Placental homogenates from infected or mock-infected dams were tested for prolactin or secretin via ELISA; control n = 12, infected n = 6. (*I*) Absorbance measurements from amniotic fluid collected from dams that were inoculated with SARS-CoV-2 vs PBS at 4 DPI: controls n = 6, infected n = 6. (*J*) Placental homogenates from either mock infected or infected were tested for SARS-CoV-2 spike protein alongside two lung homogenates from infected dams: controls n = 12, infected n = 6. For panels *A* and *B*, each data point represents one placenta from one unique mouse. In Panel *D*, each data point represents the combined RNA of 3 to 4 placentas from unique mice. (*B*, *C*, and *I*) are the summation of at least 2 independent experiments, with at least two mice per experiment. Statistical analysis was performed using an unpaired *t* test with Welsh’s correction. *P < 0.05, **P < 0.01, ***P < 0.001, and ****P < 0.0001.

First, we detected no direct SARS-CoV-2 infection of placenta via plaque assays (Fig. 2*A*). This finding was expected since there is no human ACE2 expressed outside of the maternal lung in our model. To investigate whether systemic inflammation from a mild SARS-CoV-2 infection could trigger a placental inflammatory response, we first assessed the presence of proinflammatory cytokines in placentas from infected dams. We observed a general increase in proinflammatory cytokines, with IL-10, IL-12p40, TNF, and M-CSF showing a statistically significant increase in the infected dam group compared to uninfected dams (Fig. 2*B*). Next, to assess for signs of inflammation in the placentas, a histological analysis of placentas from the same conceptuses as the cytokine analyses was performed. Although we did not observe any obvious immune infiltration, out of the 6 placentas examined from unique dams in each cohort, we observed an increase in placenta thickness in 5 out of 6 dams infected with SARS-CoV-2 compared to uninfected dams, which showed thickening in 1 out of 6 (Fig. 2*C*).

To determine the nature of inflammatory responses within the placenta, we conducted bulk transcriptomic characterization (bulk RNA-seq) of mouse placentas 4 DPI in infected and noninfected dams. Principal component analysis identified a separation between placentas from infected and noninfected dams along the first principal component (PC1) (Fig. 2*D*). Gene Ontology – Biological Process (GO-BP) pathway analysis of upregulated genes in placentas from infected dams, compared with those from control uninfected dams, revealed enrichment for select immune-related pathways, including acute-phase responses and genes involved in xenobiotic responses (Fig. 2*E*). In addition, we noted that two other distinct categories of pathways were enriched: coagulation (complement and coagulation cascade) and metabolism (vitamin transport and binding pathways). These results are consistent with prior reports that SARS-CoV-2 infection may cause a hypercoagulable state in hosts (34). Among the most upregulated genes were *Krt5*, *Muc16*, *Cyp21a1*, and *Pcdh10*, which play important roles in metabolism or cytoskeletal integrity (Fig. 2*G*) (35–39). Pathways enriched among downregulated genes in infected dam placentas included hormone activity, ribosome, and prolactin pathways (Fig. 2*G*). To determine whether transcriptomic changes in the placenta correlate with functional alterations in protein levels, we assessed the presence of developmental and metabolic hormones in the placentas of infected dams. We found that the placentas of infected dams have lower levels of secretin and prolactin, two hormones essential for fetal development (Fig. 2*H*) (40, 41).

Given above described changes in placentas from infected dams, we next examined alterations in placental barrier integrity, as maternal inflammation has been linked to placental barrier compromise and downstream effects on offspring development (42). To investigate whether there were disturbances to placental barrier integrity, Evans Blue dye was retro-orbitally injected into pregnant infected mice at 4 DPI, and the presence of Evans Blue dye in amniotic fluid was measured. Evans Blue dye has previously been reported to be a reliable indicator of placental permeability, with permeability comparable to that of albumin (43). We did not observe any statistically significant differences in Evans Blue luminescence in amniotic fluid extracted from infected versus noninfected dams, suggesting that the placental barrier remains largely unaffected by SARS-CoV-2 infection in this model (Fig. 2*H*). Finally, previous reports have suggested viral proteins may traffic to the placenta in humans long after infections (44–48). To investigate whether viral proteins in the placenta are the cause of placental abnormalities in these mice, we examined the presence of SARS-CoV-2 spike protein in placental homogenates. We found no increase in spike protein levels in placentas from infected dams relative to mock-infected controls (Fig. 2*I*). Our data indicate that maternal SARS-CoV-2 respiratory infection is sufficient to cause mild placental inflammation and disturbances, without leading to overt placental barrier compromise.

### Cerebral Cortices of Fetuses at E15 and Offspring Born to Infected Mothers Show Transcriptional Changes at P5, but Are Normalized by P30.

**Fig. 3.**
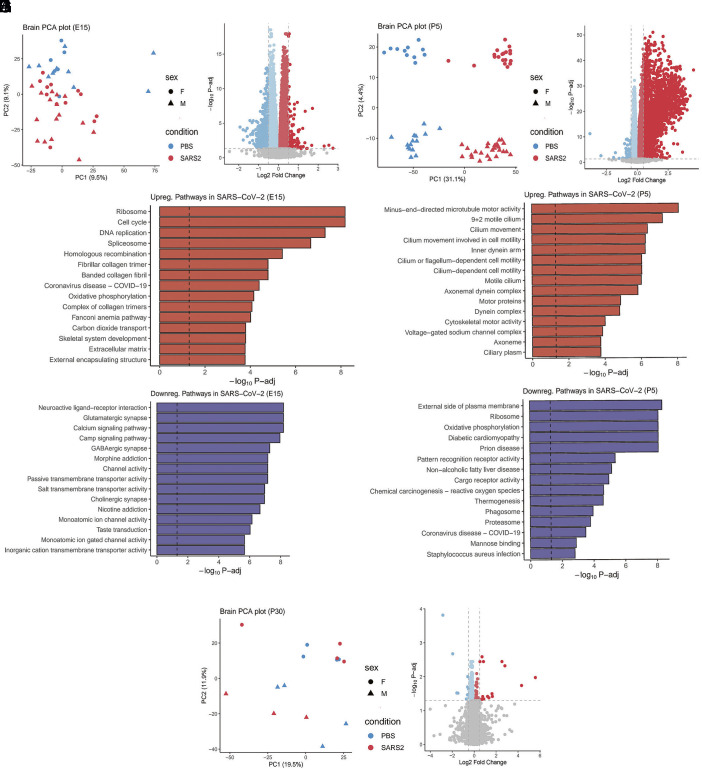
Maternal SARS-COV-2 infection induced transient transcriptomic dysregulation in the brains of offspring. (*A*) Principal component analysis of offspring brains at E15: controls n = 16 (2 litters), infected n = 28 (4 litters). (*B*) Volcano plot of differentially expressed genes at E15. (*C* and *D*) Upregulated and downregulated pathways implicated in differentially expressed genes at E15. (*E*) Principal component analysis of offspring brains at P5: controls n = 26 (4 litters), infected n = 46 (6 litters). (*F*) Volcano plot of differentially expressed genes at P5. (*G* and *H*) Upregulated and downregulated pathways implicated in differentially expressed genes at P5. (*I*) Principal component analysis of offspring brains at P30: controls n = 8 (5 litters), infected n = 7 (4 litters). (*J*) Volcano plot of differentially expressed genes at P30.

Given prior evidence of transcriptomic, cytoarchitectonic, and behavioral changes in animal models of maternal immune activation (MIA) (11, 49), we assessed whether maternal immune responses and placental inflammation in our model would affect fetal brain development. First, we performed bulk RNA-seq on microdissected cerebral cortices from offspring at E15, P5, and P30. At E15, the peak of cortical neurogenesis (50), principal component analysis identified a subtle separation in principal component (PC) 2 between offspring from infected and uninfected dams (Fig. 3*A*). GO-BP pathway analysis showed that cellular proliferation and extracellular matrix pathways were enriched in the up-regulated genes (Fig. 3*C*). Of note, at E15, extracellular matrix deposition is essential for the establishment of laminar cortical cytoarchitecture and thalamocortical circuits (51–53). Downregulated pathways included those related to synaptic development and ion channel activity (Fig. 3*D*). At P5, which is defined by the assembly of cortical circuits, principal component analysis showed a more distinct separation by infection status of the dam (PC1) and sex of the offspring (PC2) (Fig. 3*E*). At this time point, enriched pathways among upregulated genes in offspring born to infected dams included cytoskeletal and cellular motility pathways, whereas genes involved in cellular energy and metabolism were downregulated. (Fig. 3 *G* and *H*). To investigate whether the effects of maternal SARS-CoV-2 infection on the developing cortex differed by sex, we analyzed gene expression separately in male and female offspring at E15 and P5. This stratified analysis did not reveal any major sex-specific differences in the genes or pathways affected by maternal infection (*SI Appendix*, Fig. S2). However, at P30, principal component analysis did not show separation by the dam’s infection status (Fig. 3*I*). A few differentially expressed genes were identified, but they did not converge on any specific biological process (Fig. 3*J*). These data show that maternal lower respiratory tract SARS-CoV-2 infection can lead to transient transcriptomic changes in the brain, peaking at the perinatal stages and largely resolving by later childhood, in mice.

### Offspring Born to Infected Mothers Do Not Display Differences in Brain Cytoarchitecture, Microglia Number, Synaptic Density, or Behavior.

Our data showing transient transcriptomic alterations during important developmental stages led us to hypothesize that maternal lower respiratory tract SARS-CoV-2 infection may cause phenotypic changes in the neonatal brain. We first assessed gross growth deficits in the offspring and their brain. We detected no differences in the overall brain or body weight of neonatal or adult offspring (*SI Appendix*, Fig. S3 *A*–*D*).

**Fig. 4.**
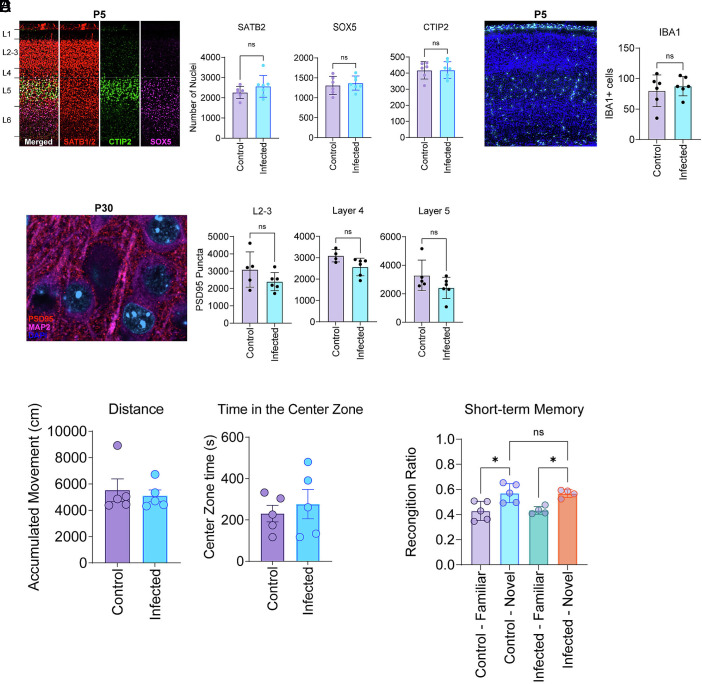
No significant differences identified in cortical cytoarchitecture or behavior after maternal SARS-CoV-2 infection. (*A*) Cortical layer and (*B*) microglia quantification of the somatosensory cortex at P5 (neonatal): controls n = 6, infected n = 6. (*C*) Synaptic puncta quantification of the somatosensory cortex at P30 (adult): controls n = 4, infected n = 6. (*D*) Results from open-field testing of adult offspring (P50) from dams either infected with SARS-CoV-2 or inoculated with PBS: controls n = 5, infected n = 5. (*C*) Results from novel object recognition of adult offspring (P50) from dams either infected with SARS-CoV-2 or inoculated with PBS: controls n = 5/4, infected n = 5/4. (*A*–*C*) is the accumulation of two independent experiments, with at least 2 pups analyzed from unique dams per experiment. D and E is from 1 independent experiment, with at least 4 pups analyzed from a total of 3 unique dams. Statistical analysis was performed using an unpaired *t* test.

Prior maternal immune activation (MIA) studies using the viral RNA mimetic poly (I:C) have shown disruptions in laminar cytoarchitecture in embryonic mice, including cortical heterotopia (11, 49). Paired with the cortical transcriptional changes at E15 and P5 converging on cell division and ECM deposition, we sought to assess for changes in cortical cytoarchitecture of neonatal offspring by quantifying the number SATB1/2 (enriched in upper layer excitatory neurons), CTIP2 (enriched in layer 5 excitatory neurons), and SOX5 (enriched in deep layer cortical neurons). In contrast to prior MIA studies, these cortical layer analyses revealed no significant differences between offspring from noninfected and SARS-CoV-2-infected dams in neonatal mice (P5) (Fig. 4*A*). Additionally, we did not identify localized heterotopias in the cortex or other gross abnormalities.

Microglia have also been investigated in the context of maternal immune activation, though with conflicting results (14, 54, 55). We found no statistically significant difference in total microglia number in the somatosensory cortex of neonatal mice (P5) born to noninfected and SARS-CoV-2 infected mothers (Fig. 4*B*). In addition, prior studies have investigated the effects of maternal infection on offspring synaptic development, and we found that synaptic pathways were downregulated at E15 (15, 56). We therefore investigated whether maternal infection had any lasting effects on long-term synaptic development. Quantification of postsynaptic densities (PSD-95) in layers 2, 3, 4, and 5 of the somatosensory cortices in adult mice (P30) revealed no significant differences between adult offspring from noninfected and SARS-CoV-2-infected mothers (Fig. 4*C*).

Finally, as numerous studies suggest that maternal infection may cause behavioral abnormalities in offspring, we conducted open-field testing and novel object recognition exams on the offspring born to either infected or noninfected dams. We selected these studies as previous studies using the MIA model identified abnormalities in these specific behavioral assays [83, 84]. For open-field testing, we measured total distance traveled and time in a designated center zone and found no significant differences between offspring born to noninfected and SARS-CoV-2-infected dams at P50 (Fig. 4*D*). Novel object recognition testing similarly found no differences in the short-term memory in offspring at P50 (Fig. 4*E*).

Together, these data demonstrate that maternally restricted SARS-CoV-2 respiratory infection can lead to transcriptomic alterations in the cerebral cortex during pre- and perinatal development. However, these alterations appear to be resolved by later postnatal developmental stages and do not result in any long-lasting gross perturbations to brain size, cortical cytoarchitecture, microglia abundance, synaptic density, or basic behavior.

## Discussion

Maternal infection has been implicated as a risk factor for neuropsychiatric disease in offspring. Considering the recent COVID-19 pandemic along with continued endemic infections, there is a crucial need to investigate the impacts of maternal SARS-CoV-2 infection on neonatal neuropsychiatric development. While clinical studies investigating the impact of maternal SARS-CoV-2 infection on early-life development have produced equivocal findings (16–20), most neuropsychiatric symptoms present during later childhood and adult stages, further underscoring the importance of animal studies to investigate potential neuropsychiatric consequences at different life stages.

Prior studies on maternal infection and offspring brain development in preclinical models have primarily utilized pathogen-associated molecular patterns (PAMPs), including poly I:C and lipopolysaccharide. These models have provided foundational biological insight into how systemic maternal innate immune activation (MIA) can affect offspring neurodevelopment. However, these models have several limitations. PAMPs induce a transient, acute, and severe immunological response, unlike the gradual response seen in natural infections. Additionally, preclinical MIA models often lead to drastic alternations in cortical cytoarchitecture, including heterotopia (11), which are rarely found in postmortem brains of patients with neuropsychiatric disorders, including autism and schizophrenia. Importantly, while there is strong evidence that severe maternal infections during pregnancy, including TORCH infections (12, 57) or those leading to hospitalization, lead to increased risk of neuropsychiatric disorders in offspring, more recent studies of mild infections during pregnancy have not identified significantly increased risk in offspring (22).

In this study, we sought to use a more physiologically relevant in vivo model of SARS-CoV-2 infection restricted to the maternal respiratory tract to investigate the impact of mild local SARS-CoV-2 infection on fetal neurodevelopment. Here, we found that maternal SARS-CoV-2 respiratory infection induced both local and systemic immune responses. We detected the increased presence of lung-resident activated CD8 T cells and SARS-CoV-2 spike protein tetramer-positive T cells as early as 4 DPI. In addition, we detected an increase in circulating proinflammatory cytokines such as TNF, CXCL10, IL-6, and IL-9 alongside circulating IgG and IgM antibodies against SARS-CoV-2 spike protein. Interestingly, although infected nonpregnant mice exhibited a proinflammatory signature at 4.5 DPI and 7 DPI, infected pregnant mice showed a diminished circulating proinflammatory cytokine signature. Of note, the serum cytokine signature in our model is modest relative to the cytokine signatures found in severe SARS-CoV-2 infection and influenza models of pregnancy (58, 59). This is consistent with the limited disease in our model. Combined with the observation that these mice displayed no overt sickness behaviors, our murine model appears to more accurately reflect the mild SARS-CoV-2 respiratory infection seen in most human patients who are pregnant.

Previous literature suggests that placental inflammation may occur in pregnant patients with SARS-CoV-2 infection despite the absence of direct placental infection (20). Similarly, in our pregnancy model of mild infection, we observed a placental inflammatory signature in infected dams, despite no direct infection of the placenta itself. Interestingly, bulk RNA-sequencing of the placenta collected at 4 DPI revealed transcriptomic upregulation of gene pathways not only related to immune function but also hypercoagulation and metabolism. Both pregnancy and SARS-CoV-2 are well known to cause a hypercoagulable state in humans (34). With our current knowledge of SARS-CoV-2 infection, both the systemic inflammation and hypercoagulability of SARS-CoV-2 infection may contribute to potential increased risk of placental disruption (60).

Interestingly, a previous study identified markers of oxidative stress, DNA damage, and complement activation in human placentas (44–48), but these were not observed in our animal data. We speculate that this difference is due to the experimental system under study. Multiple human tissues have been shown to have the capacity to support SARS-CoV-2 infection, which may affect the type and magnitude of the immune response observed (61). In addition, the previous study also observed viral proteins localizing to placental tissues, which could be hypothesized to directly trigger an immune response in the placenta with consequential damage (47). Finally, the exact infection severity status of the patients was unknown. In contrast, in our murine model, SARS-CoV-2 infection results in a mild, asymptomatic infection confined to the maternal respiratory tract. In addition to transcriptomic changes, we observed an increase in placental thickness in dams 4 d postinfection. However, we found that placental barrier function was unchanged, as we did not detect significant Evans Blue leakage into the amniotic fluid of infected dams. Future studies examining the separate mechanisms by which SARS-CoV-2-mediated inflammation and hypercoagulation cause placental dysfunction would shed light on their exact contributions to neonatal disease pathologies.

Consistent with prior literature, we observed transcriptomic alterations in the cortex at embryonic and neonatal stages but not in adults (49). However, we did not find gross abnormalities in the brains of offspring. In previous studies, cortical cytoarchitecture has been found to be disrupted in the brains of offspring of mothers injected with poly I:C (11, 49). However, in our mild SARS-CoV-2 murine pregnancy model, we did not observe any differences in laminar architecture or cell number in neonatal brains. Furthermore, we report no differences in overall brain weight in neonatal or adult offspring mice. These results suggest that maternal infection can induce transcriptomic alterations in the developing cortex of offspring, but these alterations do not lead to obvious effects on cortical structure. It is possible that the transcriptomic differences observed in the postnatal brain may reflect a response to disruption induced by maternal infection. These transient changes occurred at a developmental stage in mice analogous to human mid-fetal development – a critical period when genetic risk factors for autism and schizophrenia are known to converge (62–65). Thus, we consider a potential two-hit model for increased risk of neuropsychiatric risk in offspring requiring both maternal infection and genetic predisposition (66).

Finally, several studies have suggested that maternal SARS-CoV-2 infection can contribute to an increased risk of neurodevelopmental and psychiatric disease in the offspring. Of note, a previous research group utilized a knock-in mouse model replacing mouse ACE2 with human ACE2 to explore the consequences of prenatal infection on neurodevelopment. This model demonstrated altered hippocampal and amygdala volume, as well as sex-dependent alterations and anxiety-like behavior and locomotion (10). However, these experiments also observed significant weight loss and sickness behaviors, suggesting a more severe disease phenotype. Moreover, in this model, the central nervous system of offspring can be directly infected by SARS-CoV-2. As most SARS-CoV-2 infections are mild in nature and do not include vertical transmission of infection, the degree to which these findings can be applied to most infection cases is unclear.

This study aims to contribute to our understanding of how mild maternal SARS-CoV-2 infection can specifically affect fetal brain development. Although we observed transient transcriptomic perturbations in the offspring’s brains, we found no differences in open-field testing or novel object recognition, suggesting limited behavioral perturbations in later childhood. These results are consistent with several large cohort studies in humans, which demonstrate a limited contribution of maternal SARS-CoV-2 infection to neurodevelopmental diagnoses early in life (21–24). In addition, our results concur with a recent study by Otero et al., which suggests that an infection severity threshold exists for maternal inflammation and downstream cortical abnormalities, a finding our model did not appear to meet (58). Our results suggest that mild SARS-CoV-2 infection alone may not lead to neurodevelopmental impairment or an increased risk of psychiatric disease in infants. Mild maternal infection may contribute to neurodevelopmental or psychiatric diseases in children, but additional environmental or genetic factors may also be necessary for disease development (13, 67, 68).

There are several limitations to our study. First, we only utilized the ancestral strain (WA1) of SARS-CoV-2. Further studies using various SARS-CoV-2 strains would be informative about the extent of risk that maternal SARS-CoV-2 infection poses to developing infants. Second, our histological analysis of the brain was limited to the somatosensory cortex in a single mouse strain, similar to previous studies. Our study also only examined the consequences of maternal infection at one gestational time point. Given that different aspects of neurogenesis occur at different times during gestation, infection both earlier and later may yield different observations in our model (50). Given the compounding nature of neurogenesis disturbances, we speculate that earlier infection timing may be of interest for further evaluation, provided the dams do not undergo spontaneous abortion due to infection stress. In addition, our neurological findings were limited to transcriptional observations. Future studies employing protein-based approaches will be necessary for extending and validating our transcriptional observations. Finally, conducting experiments in ABSL3 facilities limited the scope of behavioral assays that could be conducted. A more extensive profiling of mouse behavior in this mild pregnancy infection model may reveal behavioral abnormalities that we were unable to uncover, given our limitations. Nevertheless, our study provides key insights into the transient yet significant impact of a mild, respiratory-restricted maternal infection on fetal and neonatal brain development.

## Materials and Methods

### Mouse Models.

8 to 10-week-old female C57BL6/J mice were purchased from Jackson Laboratories and housed at Yale University. All procedures used in this study followed federal guidelines and the institutional policies of the Yale School of Medicine Animal Care and Use Committee. In addition, all experiments were conducted in compliance with the BSL3 guidelines set by Yale Environmental Health & Safety.

### Cell Culture.

VeroAT cells were cultured in Gibco Dulbecco’s Modified Eagle Medium (DMEM) supplemented with 10% FBS (Gibco), 2 mM glutamine, 100 μ/mL penicillin, 100 µg/mL streptomycin (all from Gibco), and 55 µM 2-ME (Sigma-Aldrich) for all experiments.

### Viral Stock Generation.

To generate stocks of ancestral SARS-CoV-2 virus [USA-WA1/2020 (NR-52281; BEI Resources)], VeroAT cells [provided by B. Graham NIH-Vaccine Research Center (NIH-VRC)] were infected at an MOI of 0.01 for 48 h. The supernatant was clarified by centrifugation (500×*g* for 5 min) and filtered through a 0.45-μm filter. Virus containing supernatant was applied to Amicon Ultra-15 centrifugal filter (Ultracel 100 k) and spun at 2,000 rpm for 15 min prior to aliquoting and storage at −80 °C. To determine viral titers, standard plaque assays using VeroAT cells were conducted.

### Mild Maternal SARS-CoV-2 Infection Pregnancy Model.

To overexpress human ACE2 in the lower respiratory tract, female mice were infected intratracheally with AAV-hACE2 as previously described (29). Briefly, animals were anesthetized using a mixture of ketamine (100 mg/kg) and xylazine (10 mg/kg), injected intraperitoneally. The rostral neck was shaved and disinfected with iodine and ethanol. A 5-mm incision was made, the salivary glands were retracted, and the trachea was visualized. Then, a 50-μL bolus injection of AAV- hACE2 (10^11^ genome copies total) was injected into the trachea using a 500-μL insulin syringe. VetBond skin glue was used to close the incision.

After two weeks of recovery, female mice were paired with male C57BL6/J breeder and plug checked every night until detection. Following plug detection, female mice were separated from the breeder and single housed until 6 to 7 d postplug. Pregnant mice were then transferred into the BSL3 facility for acclimation.

For subsequent SARS-CoV-2 infection, pregnant female mice 10 d postplug were anesthetized using 30% vol/vol isoflurane diluted in propylene glycol. Using a pipette, 50 μL of SARS-CoV-2 (3 × 10^7^ PFU/mL) was delivered intranasally. Littermate control mice were transduced with AAV-hACE2 but inoculated with a 50-μL bolus of PBS.

### Intravascular Labeling, Cell Isolation, and Flow Cytometry.

At time points indicated, female mice were injected retro-orbitally with a 100 mL bolus of 2 μg APC-Cy7 anti-CD45 antibody for 3 min to intravascularly label circulating lymphocytes. Then, these female mice were killed and the whole left lung was harvested. The lung was cut into small pieces and digested into 3 mL of RPMI containing Collagenase A (500 μg/mL) and DNase I (12.5 μg/mL) for 40 min at 37C shaking (1,000 rpm). Lung pieces were placed onto a 70-μm filter and mashed through to create a single cell suspension. Cells were exposed to 1 mL ACK lysis buffer for 3 min and washed twice with PBS, spinning down at 500×*g* for 5 min after each wash.

The cell pellet was resuspended in 1 mL PBS and 100 μL of suspension was transferred to a 96-well plate. Single-cell suspensions were incubated with Fixable Aqua cell viability dye (Invitrogen: L34957) and anti-mouse CD16/CD32 Fc Block (BD Biosciences: 553141) for 30 min at 4 °C. Cells were washed with PBS prior to surface staining. Cells were first stained with APC-labeled SARS-CoV-2 S 62 to 76 MHC class II tetramer [I-A(b)] for 60 min at RT. Cells were washed once with PBS and then stained with anti-CD3, anti-CD44, anti-CD8a, anti-CD4, and PE-SARS-CoV-2 S 539-546 MHC class I tetramer [H-2 K(b)] for 30 min at 4 °C. After staining, cells were washed with PBS once and then fixed with 4% paraformaldehyde for 45 min at 4 °C.

Antibodies used for flow cytometry are as follows: AF700 anti-mouse CD4 (Biolegend: 100430), PerCP/Cy5.5 anti-mouse CD8a (Biolegend: 100734), BV711 anti-mouse CD44, PE SARS-CoV-2 Spike S539-546 MHC I tetramer (NIH Tetramer Core), APC/Fire 750 anti-mouse CD45 (Biolegend: 103154).

Data were acquired on an Attune NxT Flow Cytometer and analyzed by use of FlowJo Software v10.10.0. Gating strategy is provided in *SI Appendix*, Fig. S4.

### Serum Collection/Cytokine Analysis.

For all serum samples in this study, mice were anesthetized in 30% vol/vol isoflurane diluted in propylene glycol at the given timepoint. Whole blood was collected retro-orbitally and left to sit at room temperature for 15 min. Serum was isolated via centrifugation, and Triton X-100 and RNase A were added to serum samples at final concentrations of 0.5% and 0.5 mg/mL, respectively, and incubated at room temperature (RT) for 3 h before use to reduce risk from any potential virus in serum. Neutralized samples were sent to Eve Technologies for multiplex cytokine analyses (MD44). For placental homogenate analyses, whole placentas were homogenized, and Triton X-100 and RNase A were added to serum samples at final concentrations of 0.5% and 0.5 mg/mL, respectively, and incubated at room temperature (RT) for 3 h before use to reduce risk from any potential virus in the homogenates. Neutralized samples were sent to Eve Technologies for multiplex analysis (MRDMET12, MDAG16)

### Anti-SARS-CoV-2 Spike IgG and IgM ELISAs.

ELISA was conducted as previously described (29). In brief, we coated 96-well MaxiSorp plates (442404; Thermo Fisher Scientific) with 50 µL/well (final concentration of 2 µg/mL) of recombinant SARS-CoV-2 S1 protein (S1N-C52H3-100 μg; ACROBiosystems) overnight at 4 °C. Plates were then incubated for 1 h at room temperature with 200 µL of PBS containing 0.1% Tween-20 and 3% milk powder. Neutralized serum was diluted (1:50), and 100 µL of diluted serum was added for 2 h at RT. Plates were washed three times prior to addition of 50 µL of mouse IgG-specific secondary antibody (405306, 1:10,000; BioLegend) diluted in 0.1% Tween-20 and 1% milk powder to each well. Following 1 h of incubation at room temperature, plates were washed three times. Plates were developed with TMB Substrate Reagent Set (555214; BD Biosciences), and the reaction was stopped after 15 min by the addition of 2 N sulfuric acid. Plates were then read at a wavelength of 450 nm and 570 nm.

### Immunofluorescence/Histology.

For placentas, tissues were fixed with 4% PFA overnight at 4 °C followed by transfer into 30% sucrose. Fixed tissue was embedded in paraffin blocks and sectioned by the Yale Pathology Tissue Services. Hematoxylin and eosin (H&E) staining was performed by Yale Pathology Tissue Services. A blinded OB-GYN pathologist reviewed the slides and identified any pathologies. H&E images were captured using light microscopy (BX51; Olympus).

For lungs, tissues were fixed with 4% PFA overnight at 4 °C and then transferred into 30% sucrose. Lungs were embedded in OCT and sectioned at 14-μm using cryostat. Lung tissue sections were blocked in 0.1 M Tris-HCl buffer with 0.3% Triton and 1% FBS before staining. Slides were stained for E-Cadherin (DECMA-1, Thermo Fisher) and CD45 (30-F11, Biolegend) with fluorochrome-labeled primary antibody. The primary antibody rabbit anti-SARS-CoV-2 nucleocapsid (GeneTex) was used and detected with secondary antibody donkey anti-rabbit Alexa Fluor Plus 555 (Invitrogen). Slides were stained with DAPI (Sigma) and mounted with Prolong Gold Antifade reagent (Thermo Fisher). All slides were analyzed by fluorescence microscopy (BX51; Olympus) with 10x lens. Imaging data were analyzed with Imaris 7.2 (Bitplane).

For brains, tissue was fixed with 4% PFA overnight at 4 °C and then transferred into 30% sucrose. Cryopreserved brain tissue was embedded in OCT and stored at −80 °C for sectioning. Brains were sectioned on a cryostat at 50-µm thickness. Floating sections were immunostained as described previously (65). Briefly, tissue sections were washed in PBS + 0.3% Triton-X, blocked in 5% donkey serum for one hour, and then incubated with primary antibodies at 4 °C overnight. Tissue sections were then washed, incubated with secondary antibodies for 2 h at room temperature, washed, and mounted onto slides and cover-slipped with VectaShield Plus mounting media. Slides were imaged on Olympus VS200 slide scanner, and images were analyzed using FIJI (version 2.14.0/1.54f).

Cortical layer quantification was conducted semiautomatically in FIJI using a standardized threshold. Microglia quantification was conducted manually in FIJI. Synaptic density quantification was performed on IMARIS using the “Spots” tool and a standardized threshold. These analyses examined a standardized region of interest in the somatosensory cortex. Raters were blinded to the infection status of the offspring during quantification.

Antibodies used for brain immunofluorescence analysis include mouse anti-SATB1/SATB2 (Abcam: ab51502), rat anti-BCL11B (or CTIP2) (Millipore: MABE1045), rabbit anti-SOX5 (Abcam: AB94396), rabbit anti-IBA1 (FUJIFILM Wako: 019-19741), rabbit anti-PSD-95 (Invitrogen: 516900), and chicken anti-MAP2 (Novus: NB300-213).

### Spike Protein Measurement.

Placentas and lungs were homogenized using Lysing Matrix D ceramic beads, and then supernatant was cleared via centrifugation. 100 μL supernatant were treated with 2 μL 500 mM DTT (Thermo Fisher), 1 μL 100× protease inhibitor cocktail (Thermo Fisher) and 1 μL 0.5 M EDTA at 37 °C for 15 min (69). Treated tissue homogenates were used to measure spike protein concentration using a commercial ELISA kit (SinoBiological).

### Plaque Assays.

Vero AT cells were seeded into 48-well plates and left until confluence. Lung tissues were homogenized using Lysing Matrix D ceramic beads, and then supernatant was cleared via centrifugation. Supernatant was serially diluted, and 300 μL of diluted samples were overlaid onto Vero AT cells and left to incubate for 1 h at 37C, rocking every 10 min. Afterward, 0.5 mL of Avicel overlay (DMEM + 2% FBS, and 0.6% Avicel RC-581) was pipetted on top of the viral supernatant. Plaques were resolved 40 h after infection by fixing in 10% formaldehyde for 1 h followed by staining for 1 h in 0.1% crystal violet in 20% ethanol. Plates were rinsed in water to visualize plaques.

### Bulk RNA-seq/Real-Time Quantitative PCR.

For detection of SARS-CoV-2 RNA in lung tissue, approximately 33% of total lung was placed in a bead homogenizer tube with 350 μL Trizol LS (Invitrogen), and RNA was extracted with the RNeasy mini kit (Qiagen) per the manufacturer’s protocol. To quantify SARS-CoV-2 RNA levels, we used the QuantiTect Reverse Transcriptase Kit (Qiagen) with 1 μg of RNA to create total cDNA. Quantitative PCR was conducted using iTaq SYBR Green Supermix (Biorad) and the US Centers for Disease Control and Prevention real-time RT-PCR primer/probe sets for 2019-nCoV_N1.

For bulk RNA-sequencing of embryonic brains and offspring placentas, pregnant dams were killed via cervical dislocation. Embryos were harvested from the dams and kept in ice-cold HBSS. The placenta and dorsal cerebral cortex of the embryonic offspring were dissected in ice-cold HBSS. For adult offspring, the mice were euthanized via cervical dislocation, and the dorsal cortex of the brain was dissected in ice-cold HBSS. All dissected samples were then placed in Qiagen’s RNAprotect Tissue Reagent, stored at 4 °C overnight, and then transferred to −20 °C for storage. Total RNA was extracted from the samples using Qiagen’s RNeasy Mini kit. Briefly, samples were homogenized using Lysis Matrix D ceramic beads. The homogenate was centrifuged, and total RNA was extracted from the supernatant as per the kit’s instructions. RNA samples were submitted to Yale’s Center for Genome Analysis for cDNA library preparation (Kapa mRNA HyperPrep) and sequenced (NovaSeq 6000) at 100 million reads per sample.

Quality control on FASTQ reads was performed using FastQC (version 0.11.9) and a threshold set for 95% of bases meeting Q30 within a given read to ensure high sequencing quality. Paired-end reads were trimmed using Fastp (version 0.23.2) with default arguments. Pseudoalignment to mm39 mouse genome (reference provided by the Yale Center for Genome Analysis) and quantification was performed using Salmon (version 1.4.0) with selective alignment enabled. Counts were imported into R (version 4.3.2) using the tximport library (version 1.30.0). Lowly expressed genes were defined as those quantified at less than 1 count per million and were discarded from the expression matrix. Principal components analysis was performed using the prcomp function in base R. Differential expression analysis was performed using R package limma (version 3.58.1). P-values were adjusted using the Benjamini–Hochberg correction; a false discovery rate of 5% was used in detecting differentially expressed genes in brain samples and a false discovery rate of 20% was used in detecting differentially expressed genes in placental samples.

Gene symbols were converted between Ensembl IDs and Entrez IDs using the AnnotationDbi library (version 1.64.1) and bitr function (provided by clusterProfiler version 4.10.0). In pathway analysis, gene set enrichment analysis and overrepresentation analysis were performed using the clusterProfiler library (version 4.10.0), with p-value cutoff of 0.05 and q-value cutoff of 0.05. Differentially expressed genes with an absolute log-2-fold change greater than 0.5 log-2-fold change were used as input in overrepresentation analysis. In performing sex-stratified analysis, differentially expressed genes were recomputed using only reads from samples belonging to either sex condition and were used for downstream pathway analysis.

Visualizations of bulk RNA-sequencing results were created with custom scripts using the ggplot2 (version 3.5.2) library.

### Evans Blue Injections.

At E14, pregnant mice were anesthetized in 30% vol/vol isoflurane diluted in propylene glycol. 100 μL of Evans Blue dye was injected into mice retro-orbitally 15 min prior to killing. Placentas were carefully dissected out, and amniotic fluid was collected via 1 mL insulin needle. Results were read via absorbance at 620 nm on a CyTek plate reader.

### Behavioral Testing.

All behavioral assays were performed in a clean, empty rat cage (26.6 × 48.26 × 20.32 cm) covered with white plastic wrap and placed in an ABSL3 Biosafety Cabinet. Animals were handled for 2 min for 4 d before testing day. On P50, mice were subjected to open-field testing (OFT) and novel object recognition testing (NORT). To perform the OFT, the mouse was placed in the center of the rat cage and allowed to explore for 20 min. One day after the OFT, the same mouse took the NORT. The NORT is a modified version with a shortened rest window between the training and testing phase to evaluate short-term memory (<5 min) function. During the task, the mouse was placed in the center of the cage to acclimate for 10 min before being returned to the home cage for another 5 min. The mouse was then placed in the cage with two identical Lego objects for a 5-minute exploration and then returned to the home cage for 5 min. During this time, one object from the training phase was replaced with a new Lego object (of similar size, but different shape and color) for the testing phase. In the novel object testing phase, the mouse was returned to the cage and allowed to explore for 10 min. The recorded video was analyzed using Noldus analysis software (EthoVision XT 15). Only animals that explored both objects during the testing phase for an accumulation of a minimum of 20 seconds were included in the analysis.

### Statistical Analysis.

Data were analyzed using statistical tests described in the figure legends. GraphPad Prism was used to compute all statistical tests. *P* values of <0.05 were considered statistically significant.

### Graphical Illustrations.

Graphical illustrations were made with Biorender.com.

## Supplementary Material

Appendix 01 (PDF)

Dataset S01 (XLSX)

## Data Availability

Bulk RNA-seq data from Figs. 2 and 3 are available under GSE317803 (70). Computer code used to process bulk RNA-seq data and analysis is available via GitHub repository—https://github.com/caihelen/covid-pregnancy (71). All study data are included in the article and/or supporting information.
