# Supplementary material for: Mild SARS-CoV-2 maternal infection in mice induces transient offspring neurodevelopmental aberrance
Source: Proc Natl Acad Sci U S A. 2026 Mar 18;123(12):e2518294123. doi: 10.1073/pnas.2518294123 (PMC13012083; doi:10.1073/pnas.2518294123)
Supplement: Supplementary file 1 — Appendix 01 (PDF) [file pnas.2518294123.sapp.pdf]

PNAS

Supporting Information for

Mild SARS-CoV-2 maternal infection in mice induces transient offspring neurodevelopmental aberrance

Wesley Tung[1*], Matthew Yuen[2*], Helen Cai[3], Hyesun Cho[2], Peiwen Lu[1], Harvey J Kliman[4], Robert J Homer[5], Alexa Herrerias[2], Nikkita Salla[2], Arianna Rodriguez Rivera[2], Yuting Liu[2], Kartik Pattabiraman[2,3,6**], Akiko Iwasaki[1**]

1. Department of Immunobiology, Yale University School of Medicine, New Haven CT 06520, USA
2. Department of Neuroscience, Yale University School of Medicine, New Haven CT, 06520, USA
3. Child Study Center, Yale University School of Medicine, New Haven, CT, 06520, USA
4. Department of Obstetrics, Gynecology, and Reproductive Sciences, Yale University School of Medicine, New Haven, CT 06520, USA.
5. Department of Pathology, Yale University School of Medicine, New Haven, CT, 06520, USA
6. Wu Tsai Institute, Yale University, New Haven, CT, 06520, USA

* These authors contributed equally to this work.
** Co-corresponding authors.

Primary Corresponding Author: Akiko Iwasaki, akiko.iwasaki@yale.edu, ORCID: 0000-0002-7824-9856

This PDF file includes:

Figures S1 to S4

Other supporting materials for this manuscript include the following:

Datasets S1

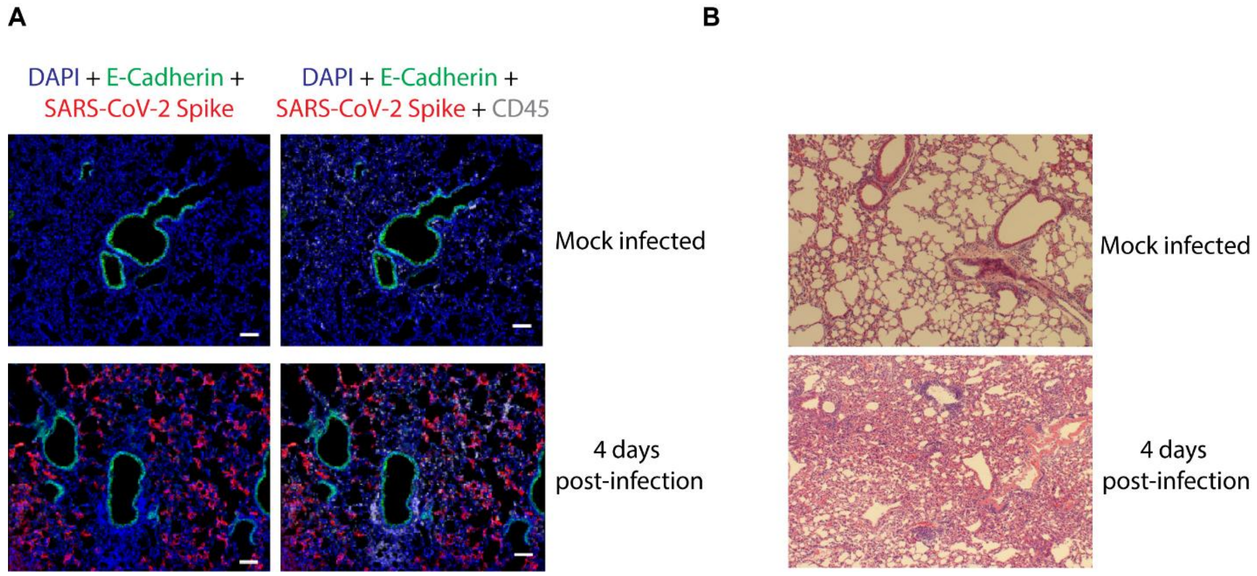

Figure S1: Whole right lung was isolated from dams either infected or mock infected with SARS-CoV-2. (A) Immunofluorescence staining to detect SARS-CoV-2 nucleocapsid protein (red), CD45+ lymphocytes (gray), E-Cadherin (green) or cell nuclei (DAPI). (B) Hematoxylin and eosin staining to detect lung pathology.

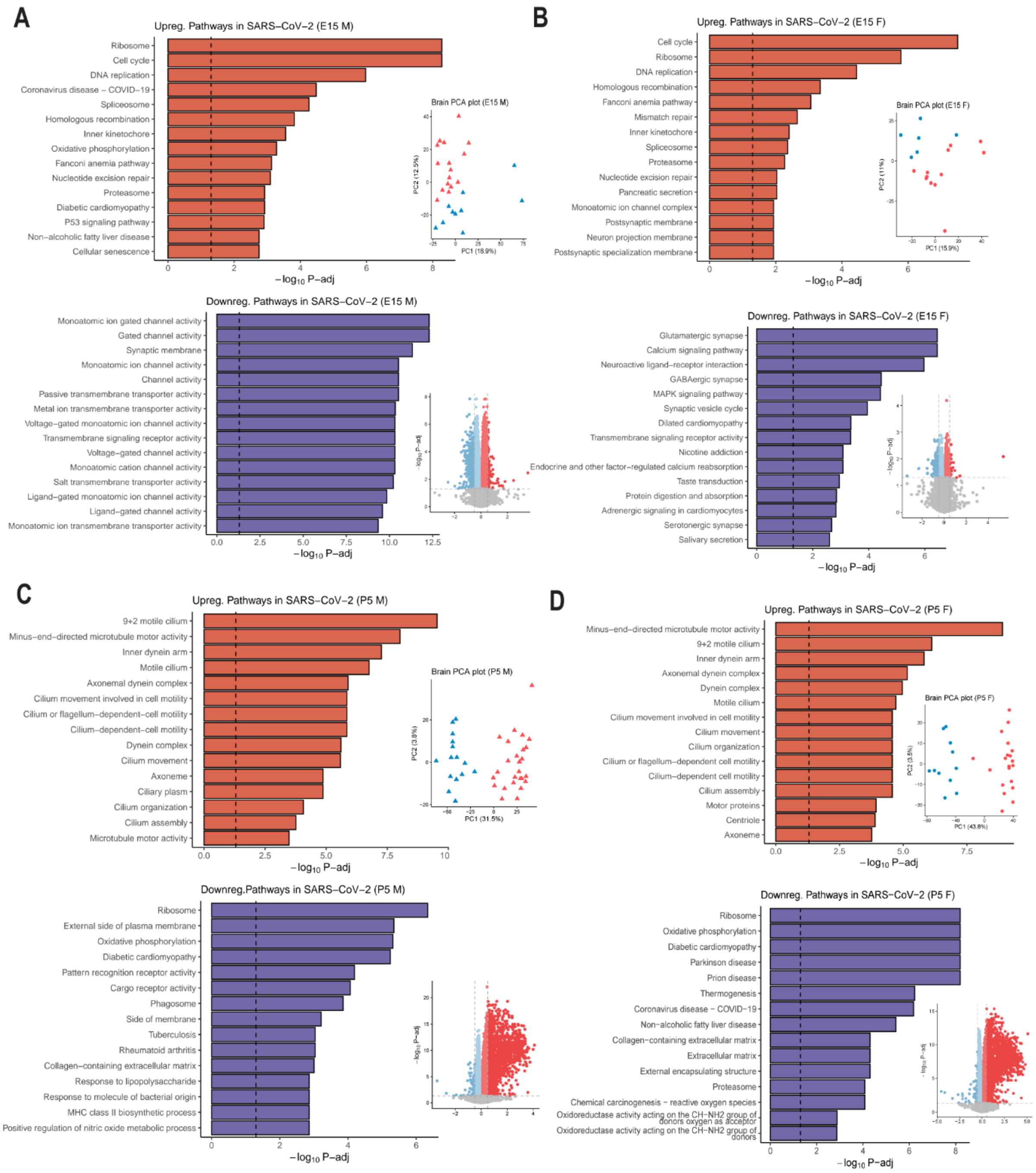

Figure S2: Transcriptomic alterations in the brain do not diverge based on sex. (A) Principal component analysis, pathway enrichment analyses, and volcano plots of E15 males: control n=10 (2 litters), infected n=16 (4 litters). (B) Principal component analysis, pathway enrichment analyses, and volcano plots of E15 females: control n=6 (2 litters), infected n=12 (4 litters). (C) Principal component analysis, pathway enrichment analyses, and volcano plots of P5 males: control n=15 (4 litters), infected n=26 (6 litters). (D) Principal component analysis, pathway enrichment analyses, and volcano plots of P5 females: control n=11 (4 litters), infected n=20 (6 litters). In all PCA plots, each data point is a single pup brain, while red/blue denotes infected dams/non-infected dams, respectively.

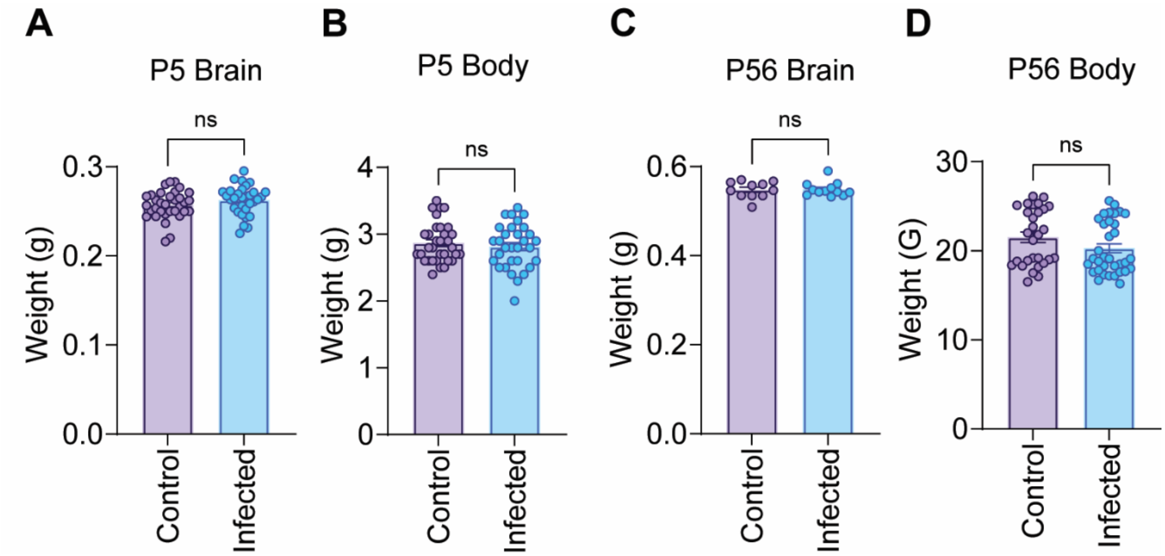

Figure S3: Maternal SARS-CoV-2 infection does not affect offspring brain or body weight. (A) P5 overall brain weight: controls n=33 (4 litters), infected n=31 (4 litters). (B) P5 overall body weight: controls n=33 (4 litters), infected n=31 (4 litters). (C) P56 overall brain weight: controls n=11 (3 litters), infected n=12 (4 litters). (D) P56 overall body weight: controls n=29 (4 litters), infected n=34 (4 litters). Statistical analysis was performed using an unpaired t test.

A

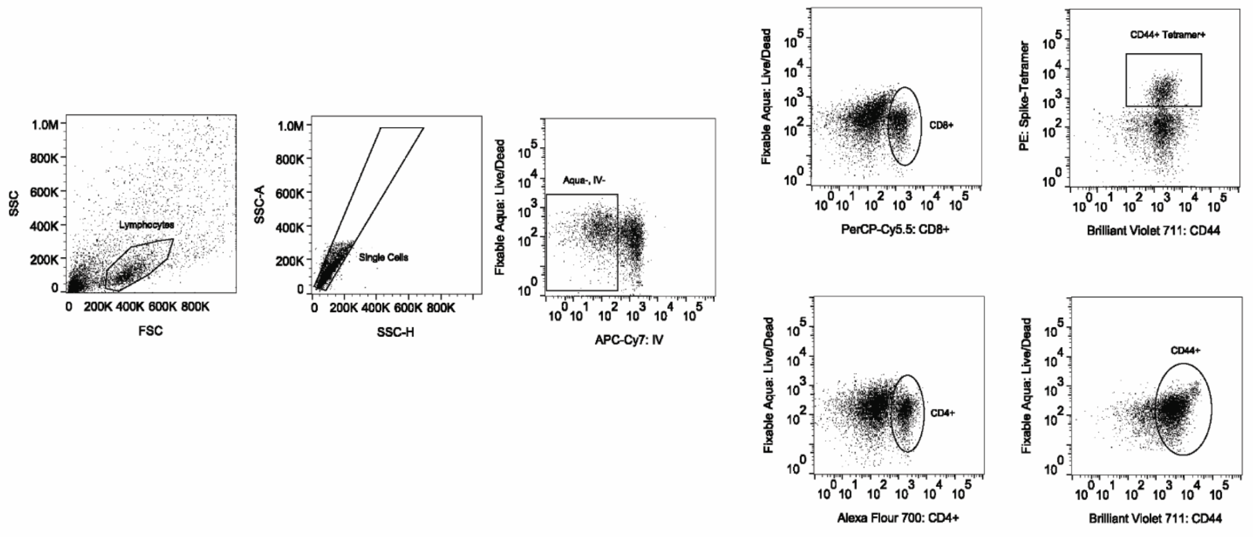

Figure S4: Flow cytometry gating scheme of T-cells in lung tissue.

Dataset S1 (separate file): Numerical values of cytokines/hormones tested in this study.
